# Association between the reflection magnitude and blood pressure in a multiethnic cohort: the Healthy Life in an Urban Setting study

**DOI:** 10.1097/HJH.0000000000003256

**Published:** 2022-08-08

**Authors:** Thomas A. Bouwmeester, Lennart van de Velde, Henrike Galenkamp, Pieter G. Postema, Berend E. Westerhof, Bert-Jan H. van den Born, Didier Collard

**Affiliations:** a Department of Vascular Medicine, Amsterdam UMC, University of Amsterdam, Amsterdam Cardiovascular Sciences, Amsterdam; b Faculty of Science and Technology, Multi-Modality Medical Imaging Group, Technical Medical Centre, University of Twente, Enschede; c Department of Public and Occupational Health, Amsterdam Public Health Research Institute, Amsterdam UMC, University of Amsterdam; d Department of Cardiology, Heart Center, Amsterdam UMC, University of Amsterdam, Amsterdam Cardiovascular Sciences; e Department of Pulmonary Medicine, Amsterdam UMC, University of Amsterdam, Amsterdam, The Netherlands

**Keywords:** blood pressure, Healthy Life in an Urban Setting, hypertension, pulse wave analysis, reflection magnitude

## Abstract

Reflection magnitude (RM), the ratio of the amplitudes of the backward and forward central arterial pressure waves, has been shown to predict cardiovascular events. However, the association with blood pressure (BP) and hypertension is unclear.

**Methods:**

We assessed RM in 10 195 individuals of Dutch, South-Asian Surinamese, African Surinamese, Ghanaian, Turkish and Moroccan origin aged between 18 and 70 years (54.2% female) participating in the Healthy Life in an Urban Setting study. To determine RM, central arterial pressure and flow were reconstructed from finger BP. Hypertension was defined based on office-BP and medication. Associations with BP, hypertension, and hypertensive organ damage were assessed using linear regression models with correction for relevant covariates.

**Results:**

Mean RM was 62.5% (standard deviation [SD] 8.0) in men and 63.8% (SD 8.1) in women. RM was lowest in Dutch and highest in South-Asian and African participants. RM increased linearly with 1.35 (95% confidence interval [CI] 1.23–1.46) for every 10 mmHg increase in systolic BP from 120 mmHg onwards, while the relation with diastolic BP was nonlinear. RM was 2.40 (95% CI 2.04–2.76) higher in hypertensive men and 3.82 (95% CI 3.46–4.19) higher in hypertensive women compared to normotensive men and women. In hypertensive men and women with ECG-based left ventricular hypertrophy or albuminuria RM was 1.64 (95% CI 1.09–2.20) and 0.94 (95% CI 0.37–1.52) higher compared to hypertensive participants without hypertensive organ damage.

**Conclusion:**

RM is associated with BP, hypertension and hypertensive organ damage, and may in part explain disparities in hypertension associated cardiovascular risk.

## INTRODUCTION

Hypertension is the leading preventable risk factor for cardiovascular morbidity and mortality worldwide [1,2]. It is well accepted that the increased arterial pulsatile load, associated with an increase in blood pressure (BP), leads to cardiovascular and renal complications [3–6]. A parameter that provides insight in the origin of the arterial pulsatile load is reflection magnitude (RM). RM is a measure of the backward pressure wave (BPW) relative to the forward pressure wave (FPW). The FPW is generated by the heart during contraction, while the BPW results from a multitude of interactions of the FPW with the arterial system. As the ratio between the amplitudes of the BPW and the FPW, RM quantifies the relative contributions of the FPW and BPW to the arterial pulse waveform. RM has shown to be an independent predictor of cardiovascular morbidity and mortality [7–10], but the relationship with BP and the presence of hypertension is still unclear.

An increase in BP may result from changes in both BPW and FPW, which may differentially affect the ratio and subsequently RM. For example, an increase in arterial stiffness and systemic vascular resistance predominantly increases BPW, whereas a higher cardiac output, mostly observed in younger hypertensive patients [11,12], predominantly affects FPW amplitude [13,14]. Earlier studies have shown important differences in the relation between hypertension and cardiovascular outcomes according to age, sex and ethnicity [15,16], these observations should be considered when investigating relationships between hypertension and RM. Furthermore, important ethnic differences in arterial pulse wave characteristics and aortic stiffness quantified using pulse wave velocity, augmentation index and aortic impedance have been described [17–20]. We hypothesized that RM is associated with BP and the presence of hypertension and associated hypertensive organ damage. Therefore, we assessed RM in relation to BP and hypertension in a multiethnic population and determined whether this relation differed according to age, sex and ethnicity.

## METHODS

### Study population and clinical outcomes

For our analysis we used baseline data collected between 2011 and 2015 of the ongoing Healthy Life in an Urban Setting (HELIUS) cohort study. A fully detailed overview of the design and methods of the HELIUS study is given in an earlier publication [21]. In brief, participants aged between 18 and 70 years living in Amsterdam, the Netherlands, were invited stratified by ethnicity (Dutch, African Surinamese, South-Asian Surinamese, Ghanaian, Turkish and Moroccan) using the municipality register. Participants were asked to bring all prescribed medication to the research visit to assess medication use. Hypertension was defined as having either an elevated office systolic BP (SBP, ≥140 mmHg); diastolic BP (DBP, ≥90 mmHg) or the use of BP-lowering medication. Medical history, including history of smoking, the presence of diabetes and incidence of cardiovascular events, was assessed using a questionnaire. Diabetes was defined as either a fasting glucose ≥7 mmol/l or the use of glucose lowering medication. Cardiovascular events were defined as either history of stroke, myocardial infarction, or coronary or peripheral revascularization. Hypertension mediated organ damage was defined as the presence of hypertension with either left ventricular hypertrophy (LVH) or increased albuminuria. Twelve-lead ECG recordings were obtained during physical examination using MAC 1600 System (GE Healthcare). All ECGs were analyzed using Modular ECG analysis System program [22] which, among other things, determines QRS complex amplitudes. Following previous publications, we defined LVH on the ECG based on the presence of at least one of three of the following electrocardiographic criteria: Sokolow–Lyon index (S V1 + R V5/V6 > 3.5 mV), the Cornell voltage (R aVL + S V3 > 2.8 mV (males), 2.0 (females) or R aVL > 1.1 mV) [23,24]. Increased albuminuria was defined as an albumin/creatinine ratio >3 mg/mmol in an early morning urine sample [25].

### Hemodynamic measurements

Peripheral BP measurements were taken twice after 5 min of resting while seated, from the left arm, using a validated, oscillometric arm device (Microlife, WatchBP Home; Microlife AG, Switzerland). The mean of these measurements was used for data analysis [21]. In 13 726 of the 22 164 participants that underwent a physical examination, continuous finger BP recordings were obtained using the Nexfin device (Edwards Lifesciences, Irvine, California, USA) [26,27]. This device measures finger BP continuously using an inflatable finger cuff, combined with an infrared plethysmograph that detects arterial pulsations. Measurements were performed for 3–5 min in supine position after 10 min of rest.

**FIGURE 1:**
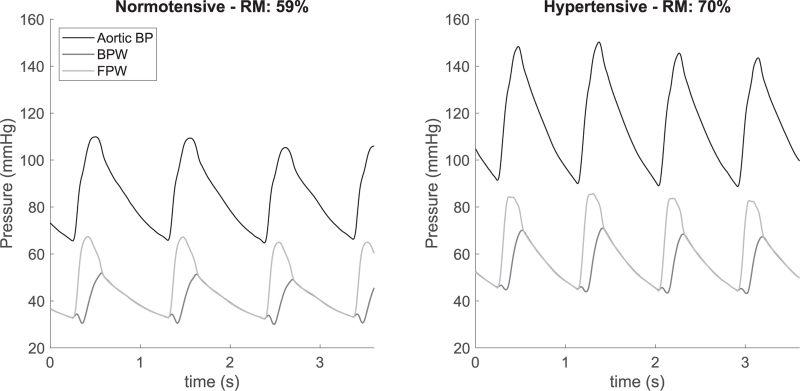
Examples of wave separation analysis. FPW and BPW are derived from the arterial central pulse wave, RM is determined as the ratio BPW/FPW × 100%. Left-hand-side is measurement from 34-year-old women with an office BP of 107/65 and a RM of 59%; right-hand-side of a 34-year-old women with an office BP of 156/92 and a RM of 70%. BP, blood pressure; BPW, backward pressure wave; FPW, forward pressure wave; RM, reflection magnitude.

Using Modelflow (version 3.7; Finapres Medical Systems, Amsterdam, The Netherlands), a continuous tracing of aortic BP was reconstructed from finger BP measurements using a generalized transfer function [28]. Aortic flow (F) was computed using a three element Windkessel model [26]. Based on these continuous variables a beat-to-beat dataset, consisting of the pressure and flow waveform and characteristic impedance of each heartbeat was derived. This dataset was further analyzed using custom written software in Matlab (R2018b, The Mathworks, Inc.). Central BP and flow were used to separate aortic BP into a forward (FPW) and a backward wave (BPW) using the following equations: FPW = (*P* + *Z* × *F*)/2; BPW = (*P* − *Z* × F)/2. An example of the measurement of RM is given in Fig. 1.

We used the characteristic impedance, *Z*, obtained from the Windkessel model during diastole. To account for noise during the measurement the original data for central pressure and flow was filtered using a Savitzky–Golay filter before reconstructing forward- and backward waves [29]. Secondly, a local moving median filter with a length of nine beats excluded all beats with a RR-interval that deviated 25% or more from the median of its preceding and following nine beats as to account for ectopic heart beats. After wave separation was carried out, the RM was calculated as the average ratio of the amplitude of the backward wave to that of the forward wave for each beat, using RM = BPW/FPW × 100%. We included all recordings of sufficient quality based on the following criteria: a minimal length of 180 s, a stable segment of least 30 beats without internal calibration of the device, sinus rhythm on the ECG, a maximum of 20% excluded beats after filtering based on variation in heart rate [30].

### Statistical analysis

Baseline characteristics of the participants, including demographics, cardiovascular risk factors, medication use, and prior cardiovascular interventions were described as %(*n*), mean (standard deviation [SD]) or median (interquartile range [IQR]) depending on the distribution of the data in individuals with and without hypertension. We used linear regression models with correction for age, sex, ethnicity for all outcomes. Based on the presence of a significant interaction between men and women in the relation between RM and hypertension status we performed separate analyses according to age category as differences between younger and older individuals appeared to be the main drivers for this interaction. RM, systolic BP, diastolic BP, BMI, FPW-amplitude and BPW-amplitude were Winsorized to the 1st and 99th percentile prior to the analysis.

As primary analysis, we determined the association of RM with systolic and diastolic BP continuously, using linear regression with RM as dependent variable. The relation between systolic- and diastolic BP and RM was modeled using sex specific restricted cubic splines, of which the order was chosen based on the Akaike information criterion. We visualized the relation with BP using predicted values from the model for the Dutch ethnicity, an age of 35 years for younger participants, an age of 65 years for older participants and an age of 50 years when looking at the overall cohort. Secondly, we assessed the relation of RM with history of hypertension. We performed a sensitivity analysis in participants without BP-lowering drugs. To elucidate whether differences in RM were caused by changes in FPW, BPW or both, we assessed the association between the FPW-amplitude and BPW-amplitude with hypertension separately. Thirdly, we assessed the relation with hypertensive mediated organ damage by comparing RM in normotensive participants to participants with hypertension as well as to participants with hypertension and LVH or increased albuminuria.

All analysis were performed using a model with correction for sex, age and ethnicity, and if applicable with an interaction term for sex and ethnicity. Secondly, we repeated all our analyses with additional correction for height. The results from the regression models were expressed as percentage points quantifying absolute changes in RM. All statistical analyses were performed with R version 4.0.2, figures were created using ggplot2 version 3.3.3. *P* values <0.05 were considered significant.

## RESULTS

### Baseline characteristics

We analyzed a total of 10 195 participants with continuous BP recordings of sufficient quality (Figure 1, Supplemental Digital Content). Median age was 46 years [IQR 33–54], 54.2% were female. Hypertensive participants were generally older (53 years [IQR 47–59] vs. 40 years [IQR 29–59], *P* < 0.001), more often of Ghanaian or African Surinamese descent, had a higher prevalence of diabetes (21.1 vs. 4.1%, *P* < 0.001) and history of cardiovascular disease (13.0 vs. 5.7%, *P* < 0.001) compared to normotensive participants. In addition, BMI was higher in hypertensive (mean 29.7 kg/m^2^, SD 5.0) compared to normotensive participants (26.4 kg/m^2^, SD 4.8) (*P* < 0.001) and average height was lower in hypertensive compared to normotensive men and women. (168 ± 10.0 cm vs. 169 ± 10.1 cm, *P* < 0.001). Antihypertensive medication was prescribed in 49.2% of hypertensive participants. The prevalence of ECG-based LVH was higher in hypertensive men (40.7%) compared to hypertensive women (30.4%) (*P* < 0.001), whereas the prevalence of albuminuria was similar between hypertensive men and women (9.3 and 10.4%, *P* = 0.32). The overall prevalence of hypertension was 22.8% in men and 15.7% in women aged below 50 years and 58.6% in men, and 55.3% in women above 50 years of age. A complete overview of the baseline characteristics stratified by age and sex is given in Table 1.

**TABLE 1:** Baseline characteristics; stratified by age (<50 and ≥50 years) and sex

	Age < 50		Age ≥ 50	
	Men	Women	Men	Women
*n*	2759	3436	1911	2089
Age	37 [28, 44]	36 [27, 44]	56 [53, 61]	56 [52, 60]
Ethnicity				
Dutch	593 (21.5)	527 (15.3)	491 (25.7)	423 (20.2)
SAS	410 (14.9)	352 (10.2)	270 (14.1)	298 (14.3)
AS	405 (14.7)	593 (17.3)	446 (23.3)	620 (29.7)
Ghanaian	267 (9.7)	445 (13.0)	274 (14.3)	258 (12.4)
Turkish	604 (21.9)	699 (20.3)	213 (11.1)	205 (9.8)
Moroccan	480 (17.4)	820 (23.9)	217 (11.4)	285 (13.6)
Smoking	937 (34.1)	668 (19.5)	542 (28.5)	340 (16.3)
Systolic BP (mmHg)	127.6 (13.7)	119.2 (15.1)	137.0 (16.4)	134.3 (17.9)
Diastolic BP (mmHg)	80.2 (9.6)	74.5 (10.1)	84.9 (9.9)	79.8 (9.9)
BMI (kg/m^2^)	26.2 (4.1)	27.3 (5.6)	27.2 (3.9)	30.0 (5.4)
Height (cm)	177 (7.8)	163 (7.2)	174 (7.8)	161 (7.1)
Total cholesterol (mmol/l)	4.84 (1.01)	4.65 (0.90)	5.09 (1.09)	5.24 (1.08)
Diabetes	115 (4.2)	112 (3.3)	365 (19.2)	412 (19.8)
History of CV-event	88 (3.2)	64 (1.9)	210 (11.1)	139 (6.8)
Hypertension	629 (22.8)	541 (15.7)	1119 (58.6)	1155 (55.3)
Albuminuria	178 (6.5)	408 (11.9)	217 (11.4)	203 (9.7)
LVH	1228 (44.5)	501 (14.6)	628 (32.9)	477 (22.8)
BP-lowering drugs	135 (4.9)	248 (7.2)	548 (28.7)	764 (36.6)

Data are presented as mean (SD) or *n* (%). AS, African Surinamese; BMI, body mass index; BP, blood pressure; CV-event, cardiovascular event; LVH, left ventricular hypertrophy on ECG; SAS, South-Asian Surinamese.

### Reflection magnitude is associated with systolic and diastolic BP

**FIGURE 2:**
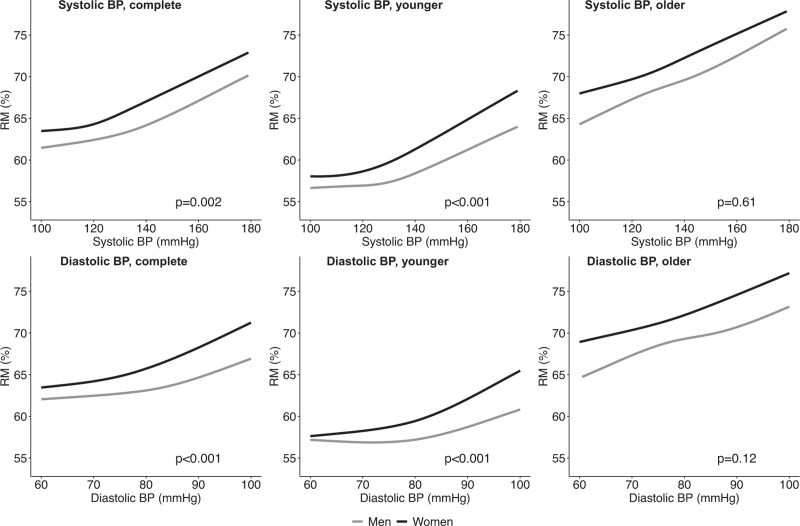
Relationship between systolic and diastolic blood pressure and reflection magnitude in the complete cohort, in younger (<50 years) and older (≥50 years) participants. Lines represent the results from regression model with correction for age, sex, ethnicity, depicted with respect to age of 35 years in younger cohort; 65 in older cohort and 50 in the complete cohort; for the Dutch ethnicity. BP, blood pressure; RM, reflection magnitude.

RM was 62.5% (SD 8.0) in men and 63.8% (SD 8.1) in women. In both men and women, RM increased linearly by 1.35 (95% CI 1.24–1.46) per 10 mmHg increase in SBP between 120 mmHg and 180 mmHg (Fig. 2). The relation with DBP showed an increase of the slope above 80 mmHg, with a 1.99 (95% CI 1.61–2.38) increase per 10 mmHg rise in DBP for the overall population. In individuals below 50 years of age, we observed an inflection point around 130 mmHg for SBP, after which RM increased linearly with 1.22 (95% CI 0.90–1.54) and 1.62 (95% CI 1.25–1.99) per 10 mmHg rise in SBP in men and women, respectively. For DBP, RM increased linearly by 1.55 (95% CI 0.88–2.21) per 10 mmHg above DBP 80 mmHg in men and by 3.03 (95% CI 2.26–3.81) per 10 mmHg in women. In older adults, we found no significant interaction with sex (*P* = 0.61), despite the overall higher RM in women. Here, the relation with RM was linear across the complete BP range, increasing by 1.32 (95% CI 1.20–1.45) and 1.90 (95% CI 1.65–2.15) for every 10 mmHg in SBP and DBP, respectively.

The regression analyses showed that the explained variance of RM by SBP, age, sex and ethnicity was 54% in the complete group, 44% in the younger subgroup and 25% in the older subgroup. RM increased significantly with age (0.36 per year; 95% CI 0.35–0.37). All ethnicities, except for those of Moroccan origin, had a significantly higher RM as compared to Dutch, ranging from 0.71 (95% CI 0.35–1.08) in Turkish participants, to 2.02 (95% CI 1.63–2.41) in South-Asian Surinamese participants. Following correction for height, the relation with SBP remained similar, however ethnic differences were attenuated. There were no significant differences between South-Asian Surinamese, African Surinamese and Ghanaian participants, compared to Dutch while RM in Turkish and Moroccan participants was significantly lower (Table 1, Supplemental Digital Content). There was no interaction between BP and ethnicity, except for DBP in the older subgroup (*P* = 0.031; Figure 2, Supplemental Digital Content).

### Reflection magnitude is associated with hypertension, mainly through alterations in backward pressure wave reflection

RM was significantly associated with the presence of hypertension (*P* < 0.001), with a significant interaction with sex in the crude model (Table 2). RM was 3.00 (95% CI 2.64–3.36) higher in hypertensive compared to normotensive women, and 1.98 (95% CI 1.61–2.34) higher in hypertensive compared to normotensive men. Normotensive women had a 1.60 (95% CI 1.31–1.88) higher RM compared to normotensive men in the crude model. Following correction for height, RM was lower in normotensive women compared to men, the relation with hypertension remained similar. The interaction with sex was mainly driven by younger hypertensive individuals, in older adults we found no interaction with sex (*P* = 0.60).

**TABLE 2:** Regression analysis showing the association between reflection magnitude and the presence of hypertension, with correction for age, sex and ethnicity

		Complete				Younger (<50)				Older (≥50)			
	Term	Est.	95% CI		*P*-value	Est.	95% CI		*P*-value	Est.	95% CI		*P*-value
Age, ethnicity	NT	0.00	Ref			0.00	Ref			0.00	Ref		
	HT	1.98	1.61	2.34	<0.001	1.92	1.45	2.38	<0.001	2.34	1.90	2.78	<0.001
	Women	1.60	1.31	1.88	<0.001	1.37	1.08	1.65	<0.001	2.31	1.89	2.73	<0.001
	Interaction HT × women	1.02	0.54	1.50	<0.001	1.69	1.02	2.35	<0.001	–	–	–	–
Age, ethnicity, height	NT	0.00	Ref			0.00	Ref			0.00	Ref		
	HT	2.00	1.65	2.36	<0.001	2.05	1.60	2.51	<0.001	2.33	1.89	2.76	<0.001
	Women	−0.63	−0.99	−0.27	<0.001	−1.03	−1.41	−0.64	<0.001	0.29	−0.32	0.89	<0.001
	Interaction HT^∗^ women	1.01	0.54	1.48	<0.001	1.58	0.94	2.22	<0.001	−	−	−	−

Interaction term depicts interaction between sex and hypertension; estimates are depicted with men as reference. Interaction analysis did not show significant impact of ethnicity on the relation between HT and RM. CI, confidence interval; Est, estimate; HT, hypertension; NT, normotension; ref, reference.

In normotensive women, the elevated RM was mainly driven by a lower FPW amplitude of 1.48 (95% CI 1.18–1.78) mmHg compared to men, while BPW was only 0.36 (95% CI 0.14–0.58) mmHg lower. BPW was 0.55 (95% CI 0.25–0.85) mmHg higher in hypertensive women compared to men, while FPW amplitude was comparable (−0.40, 95% CI −0.81–0.02, *P* = 0.06). Sex differences were attenuated following correction for height, however there remained a significant interaction between hypertension, FPW and BPW (Table 2, Supplemental Digital Content).

### Reflection magnitude is associated with hypertension mediated organ damage

RM was 1.64 (95% CI 1.09–2.20) higher in men with increased albuminuria and/or LVH compared to hypertensive men without hypertensive organ damage. Compared to normotensive individuals, RM was 2.88 (95% CI 2.40–3.35) higher if albuminuria or LVH was present. In female participants with hypertension and either albuminuria or LVH, RM was 0.94 (95% CI 0.37–1.52) higher compared to hypertensive women without hypertensive organ damage and 3.61 higher (95% CI 3.09–4.13) compared to normotensive women. These relations did not materially change following additional correction for height (Table 3).

**TABLE 3:** Regression analysis showing the association between reflection magnitude and hypertension, left ventricular hypertrophy and increased albuminuria

		Complete				Younger (<50)				Older (≥50)			
	Term	Est.	95% CI		*P*-val	Est.	95% CI		*P*-val	Est.	95% CI		*P*-val
Age, ethnicity	NT	0.00	Ref			0.00	Ref			0.00	Ref		
	HT	1.23	0.79	1.67	<0.001	1.13	0.51	1.74	<0.001	1.82	1.34	2.31	<0.001
	HT + LVH/IA	2.88	2.40	3.35	<0.001	2.73	2.11	3.34	<0.001	3.19	2.62	3.75	<0.001
	Women	1.60	1.32	1.88	<0.001	1.37	1.08	1.66	<0.001	2.38	1.96	2.80	<0.001
	HT ∗ women	1.44	0.86	2.02	<0.001	2.16	1.31	3.00	<0.001	–	–	–	–
	(HT + LVH/IA) × Women	0.74	0.07	1.41	0.031	1.47	0.52	2.41	0.002	–	–	–	–
			Overall	<0.001		Overall	<0.001		Overall	0.32			
Age, ethnicity, height	NT	0.00	Ref			0.00	Ref			0.00	Ref		
	HT	1.26	0.83	1.70	<0.001	1.25	0.65	1.86	<0.001	1.81	1.33	2.30	<0.001
	HT + LVH/IA	2.89	2.43	3.36	<0.001	2.86	2.26	3.46	<0.001	3.18	2.62	3.73	<0.001
	Women	−0.63	−0.99	−0.27	<0.001	−1.03	−1.42	−0.65	<0.001	0.35	−0.25	0.95	0.253
	HT × women	1.40	0.83	1.97	<0.001	2.01	1.18	2.84	<0.001	–	–	–	–
	(HT + LVH/IA) × women	0.78	0.12	1.44	0.020	1.44	0.52	2.37	0.002	–	–	–	–
			Overall		<0.001		Overall		<0.001		Overall		0.40

Interaction term depicts interaction between sex and hypertension; estimates are depicted with men as reference. There was no interaction between ethnicity and the presence of hypertension mediated organ damage. CI, confidence interval; Est, estimate; HT, hypertension; IA, increased albuminuria; LVH, left ventricular hypertrophy on ECG; NT, normotension; ref, reference.

## DISCUSSION

We show that there is a consistent and linear relation between RM and both systolic and diastolic BP and that RM is independently associated with hypertension, and hypertension mediated organ damage, mainly through an increase in BPW-amplitude. We extend these observations by showing that RM is associated with BP and that there are important differences between sexes and across ethnic groups. This is in line with previous observations, which shows that cardiovascular risk differs in men and women with different hypertensive phenotypes and across ethnic groups [31–33].

Our study confirms earlier findings from the Heinz Nixdorf Recal study, which showed that RM increases with age and is larger for women than men [34]. In addition, we observed differences in the relation between RM and hypertension in men and women <50 years of age suggesting that younger women are more severely affected by the increased arterial load than men. This was also supported by our continuous analysis, which showed that the slope between RM and SBP was steeper in younger women compared to younger men.

In contrast, we did not observe an interaction in the older age group. This could be attributable to the increase in arterial stiffness that comes with age in both sexes [35]. In line, we found nonlinearities in the continuous analysis of RM and BP in younger adults and a lower explained variance by BP. This suggests that in younger individuals with a lower characteristics impedance large changes in BP are required to develop adverse wave reflections [36]. In these participants, we observed a steep nonlinear increase in BP values above 130/80 mmHg, whereas we observed a linear relation across the complete range of BP in older adults.

The higher RM in normotensive women as compared to men was primarily the result of a lower FPW, and disappeared after correction for height. It is well known that left ventricular size and stroke volume are lower in women as compared to men [37,38], but correction for height did not account for all differences in FPW amplitude. We observed that BPW amplitude was higher in hypertensive women compared to hypertensive men, whereas it was significantly lower in normotensive women compared to normotensive men. Correction for height attenuated differences in RM between normotensive men and women, however the interaction term did not materially change. This suggests that in hypertensive women wave reflection is higher compared to hypertensive men. Indeed previous studies have shown that aortic characteristic impedance is higher and arterial compliance was lower in women compared to men [39,40]. The sex differences in the relation with hypertension are in apparent contrast with the results by Chester *et al.*[41], who showed that RM was not associated with heart failure in women, while there was a significant association in men. However they did not use sex-specific values for the derivation of quantiles of RM in their analysis, which may be relevant given the different RMs for men and women [42].

RM was higher in all ethnic groups compared to the Dutch reference population, with the highest RM in African and South-Asian descent populations. This is in line with previous observations by us and others concerning other measures of wave reflection, including augmentation index [19,20]. After correction for body height, however, differences in RM between the different ethnic groups were significantly attenuated, suggesting that body height explains a large part of the variation in RM across ethnic groups. However, the relation with hypertension remained unchanged, indicating that hypertension is related to different physiological changes that result in increased BPW reflections and higher RM.

Our findings may partly explain differences in the effect of hypertension on the cardiovascular system that is mediated by differences in the relative contributions of the FPW and BPW. The increase in afterload, posed by the increase in BPW amplitude could be a possible explanation why participants of African descent in particular show more hypertension related complications [32]. This is further supported by our finding that RM was higher in hypertensive individuals with increased albuminuria, LVH on ECG or both, supporting earlier findings by Weber *et al.*[9]. In addition, the increase in RM may directly contribute to structural cardiac changes and LVH. Myocardial wall stress (MWS) is dependent on arterial load, and time-varying left ventricular geometry [43,44]. In people with preserved ejection fraction, peak MWS occurs at early systole, after which MWS decreases, while left ventricular pressure is maintained. This shift between the relationship of MWS and left ventricular pressure seems to be protective against an increase in ventricular mass [43]. In patients with concentric remodeling of the ventricle, as is the case in LVH, this shift in MWS and LV-pressure relationship is less pronounced, causing a delay in peak MWS from early systole, to mid and late systole. This is the period in which the left ventricle is exposed to major wave reflections [45]. The exposure of the ventricle to wave reflections during peak MWS, in combination with higher RM, might predispose to increased LV mass. This is supported by the analysis of Chirinos *et al.*[7], which shows that RM was strongly predictive of new onset heart failure, while associations with other cardiovascular events were smaller in effect size. Identifying patients at risk because of excessive wave reflection may therefore be helpful as it forms a potential target for the prevention of complications [46].

Strengths of our analysis include the large-scale automatic analysis of hemodynamic measurements in a multiethnic population-based cohort. This allowed us to assess RM in over 10 000 men and women over a wide age range and with different ethnic backgrounds. In addition, the combination of continuous finger BP measurements, which are relatively easy to perform, combined with automatic analysis allowed us to determine RM with small interobserver variability. An advantage of RM is that it is less influenced by other hemodynamic variables including heart rate, left ventricular contractility and preload compared with the augmentation index [7,47,48]. Limitations of the study include the use of a generalized transfer function to estimate central aortic pressure and a three-element Windkessel model for determination of the flow wave, which are used to estimate central forward- and backward wave amplitude, upon which the RM value relies. The use of a generalized transfer function is a commonly used technique to estimate central aortic pressure [28,49]. These techniques haves been validated previously, but in mainly older populations [50,51]. LVH was assessed based on ECG criteria, which have relatively high specificity, but low sensitivity compared to ultrasound or cardiac magnetic resonance techniques [52]. Still, we found much higher numbers of participants with ECG-LVH than is to be expected in a general population. This indicates that the reported relatively high specificity of the ECG criteria for LVH are probably an overestimation. We tried to mitigate this effect by using composite LVH on ECG criteria, as suggested in earlier studies [23,24]. Finally, hypertension was defined based on office BP-measurements following current ESH recommendations [1], which did not allow us to exclude possible white coat effects when identifying individuals with hypertension.

In conclusion, we show that there is a consistent relation between RM and BP that depends on age and sex, and varies across ethnic groups. Expanding on these findings, we show that RM is associated with hypertension and that the presence of albuminuria and LVH. Interestingly, body height was an important determinant of ethnic and sex differences in wave reflection, but the relation with BP remained virtually unchanged. This suggests that the reflection magnitude, as a pressure independent ratio of wave reflection, may offer further predictive value above and beyond BP alone in predicting cardiovascular outcomes in younger and older adults with hypertension. Prospective data can aid in disentangling the causal role of RM in the development of sustained hypertension and its predictive value for the development of cardiovascular complications in individuals with and without hypertension.

## ACKNOWLEDGEMENTS

We would like to thank the participants, research nurses and HELIUS staff for their help in data collection.

Funding: The Academic Medical Center (AMC) of Amsterdam and the Public Health Service of Amsterdam (GGD) provided core financial support for HELIUS. The HELIUS study is also funded by research grants of the Dutch Heart Foundation [Hartstichting; 2010T084], the Netherlands Organization for Health Research and Development [ZonMw; 200500003], the European Integration Fund [EIF; 2013EIF013], and the European Union [Seventh Framework Programme, FP-7; 278901].

Author contributions: T.A.B., D.C. and Prof. B.-J.H.vdB. designed the original study. T.A.B., and D.C. performed data and statistical analysis. T.A.B., D.C. and B,-J.H.vdB. drafted the paper. T.A.B., L.vdV., H.G., P.G.P., B.-J.vdB. and D.C revised the paper. All authors approved the final version of the manuscript.

### Conflicts of interest

There are no conflicts of interest.

## Supplementary Material

Supplemental Digital Content
