# Supplementary material for: Association between the reflection magnitude and blood pressure in a multiethnic cohort: the Healthy Life in an Urban Setting study
Source: J Hypertens. 2022 Aug 8;40(11):2263–70. doi: 10.1097/HJH.0000000000003256 (PMC9553245; doi:10.1097/HJH.0000000000003256)
Supplement: Supplemental Digital Content [file jhype-40-2263-s001.doc]

|  |  | **Complete** | | | | | | | **Younger (<50)** | | | | | | | **Older (≥ 50)** | | | | | | |
| --- | --- | --- | --- | --- | --- | --- | --- | --- | --- | --- | --- | --- | --- | --- | --- | --- | --- | --- | --- | --- | --- | --- |
| SBP | **term** | **Est.** | **95%CI** | | | | **p-val** | | **Est.** | **95%CI** | | | | **p-val** | | **Est.** | **95%CI** | | | | **p-val** | |
| Crude | SAS | 2.02 | 1.63 | | 2.41 | | <0.001 | | 2.48 | 2.01 | | 2.94 | | <0.001 | | 1.33 | 0.64 | | 2.01 | | <0.001 | |
|  | AS | 0.75 | 0.40 | | 1.10 | | <0.001 | | 1.38 | 0.94 | | 1.81 | | <0.001 | | -0.11 | -0.69 | | 0.47 | | 0.718 | |
|  | Ghanaian | 1.71 | 1.30 | | 2.12 | | <0.001 | | 2.30 | 1.82 | | 2.79 | | <0.001 | | 0.59 | -0.13 | | 1.31 | | 0.107 | |
|  | Turkish | 0.71 | 0.35 | | 1.08 | | <0.001 | | 1.02 | 0.62 | | 1.43 | | <0.001 | | 0.19 | -0.58 | | 0.95 | | 0.631 | |
|  | Moroccan | -0.11 | -0.47 | | 0.26 | | 0.569 | | 0.29 | -0.12 | | 0.69 | | 0.163 | | -0.77 | -1.48 | | -0.06 | | 0.033 | |
|  | Age | 0.35 | 0.34 | | 0.36 | | <0.001 | | 0.35 | 0.34 | | 0.37 | | <0.001 | | 0.27 | 0.23 | | 0.31 | | <0.001 | |
| Full | SAS | 0.06 | | -0.37 | | 0.49 | | 0.788 | 0.45 | | -0.05 | | 0.94 | | 0.079 | -0.58 | | -1.35 | | 0.20 | | 0.146 |
|  | AS | -0.31 | | -0.67 | | 0.05 | | 0.092 | 0.17 | | -0.27 | | 0.62 | | 0.441 | -1.05 | | -1.66 | | -0.45 | | <0.001 |
|  | Ghanaian | 0.01 | | -0.43 | | 0.44 | | 0.978 | 0.41 | | -0.10 | | 0.93 | | 0.114 | -0.97 | | -1.74 | | -0.19 | | 0.015 |
|  | Turkish | -1.28 | | -1.69 | | -0.87 | | <0.001 | -1.03 | | -1.48 | | -0.59 | | <0.001 | -1.89 | | -2.75 | | -1.03 | | <0.001 |
|  | Moroccan | -1.77 | | -2.16 | | -1.38 | | <0.001 | -1.47 | | -1.90 | | -1.04 | | <0.001 | -2.37 | | -3.14 | | -1.60 | | <0.001 |
|  | Age | 0.32 | | 0.31 | | 0.33 | | <0.001 | 0.33 | | 0.32 | | 0.35 | | <0.001 | 0.23 | | 0.19 | | 0.27 | | <0.001 |
|  | Height | -0.18 | | -0.19 | | -0.16 | | <0.001 | -0.19 | | -0.21 | | -0.17 | | <0.001 | -0.16 | | -0.20 | | -0.13 | | <0.001 |

**Supplementary table 1:** Coefficients of regression model for relationship between SBP and RM. All models included additional correction for sex, spline term for SBP, and an interaction term between sex and SBP, coefficients are not shown. (Est = Estimate, CI = confidence interval, SAS = South-Asian Surinamese, AS = African Surinamese).

|  |  | **Complete** | | | | | **Younger (<50)** | | | | | | **Older (≥ 50)** | | | | |
| --- | --- | --- | --- | --- | --- | --- | --- | --- | --- | --- | --- | --- | --- | --- | --- | --- | --- |
|  | **term** | **Est.** | **95%CI** | | **p** | | **Est.** | **95%CI** | | | **p** | | **Est.** | **95%CI** | | | **p** |
|  | NT | 0.00 | Ref |  | |  | 0.00 | | Ref |  | |  | 0.00 | | Ref |  |  |
| Age, ethnicity | HT | 2.61 | 2.22 | 3.01 | | <0.001 | 2.16 | | 1.66 | 2.66 | | <0.001 | 3.52 | | 3.01 | 4.04 | <0.001 |
|  | Women | 1.61 | 1.35 | 1.88 | | <0.001 | 1.37 | | 1.08 | 1.65 | | <0.001 | 2.41 | | 1.92 | 2.90 | <0.001 |
|  | Interaction: HT-Women | 1.44 | 0.85 | 2.03 | | 0.000 | 1.93 | | 1.14 | 2.71 | | <0.001 | - | | - | - | - |
| Age, ethnicity, height | NT | 0.00 | Ref |  | |  | 0.00 | | Ref |  | |  | 0.00 | | Ref |  |  |
|  | HT | 2.69 | 2.31 | 3.08 | | <0.001 | 2.33 | | 1.85 | 2.82 | | <0.001 | 3.26 | | 2.58 | 3.94 | <0.001 |
|  | Women | -0.74 | -1.09 | -0.39 | | <0.001 | -1.10 | | -1.48 | -0.72 | | <0.001 | 0.05 | | -0.73 | 0.83 | 0.898 |
|  | Interaction: HT-Women | 1.34 | 0.76 | 1.92 | | <0.001 | 1.81 | | 1.04 | 2.57 | | <0.001 | - | | - | - | - |

**Supplementary table 2:** Regression analysis showing the relationship between RM and HT in the population using no anti-hypertensive medication. Interaction term depicts interaction between sex and hypertension; estimates are depicted with men as reference (Est = Estimate, NT = normotension, HT = hypertension, ref = reference, CI = confidence interval).

|  |  | **Complete** | | | | **Younger (<50)** | | | | **Older (≥ 50)** | | | |
| --- | --- | --- | --- | --- | --- | --- | --- | --- | --- | --- | --- | --- | --- |
| FPW | **term** | **Est.** | **95%CI** | | **p** | **Est.** | **95%CI** | | **p** | **Est.** | **95%CI** | | **p** |
|  | NT | 0.00 | Ref |  |  | 0.00 | Ref |  |  | 0.00 | Ref |  |  |
| Age, ethnicity | HT | 2.53 | 2.14 | 2.91 | <0.001 | 2.00 | 1.47 | 2.52 | <0.001 | 3.15 | 2.71 | 3.59 | <0.001 |
|  | Women | -1.48 | -1.78 | -1.18 | <0.001 | -1.98 | -2.30 | -1.65 | <0.001 | -0.30 | -0.72 | 0.12 | 0.161 |
|  | Interaction HT-Women | 1.08 | 0.57 | 1.59 | 0.000 | 1.72 | 0.98 | 2.46 | <0.001 | - | - | - | - |
| Age, ethnicity, height | NT | 0.00 | Ref |  |  | 0.00 | Ref |  |  | 0.00 | Ref |  |  |
|  | HT | 2.52 | 2.13 | 2.90 | <0.001 | 1.93 | 1.41 | 2.45 | <0.001 | 3.15 | 2.72 | 3.59 | <0.001 |
|  | Women | -0.62 | -1.01 | -0.23 | 0.002 | -0.79 | -1.23 | -0.35 | <0.001 | 0.23 | -0.38 | 0.83 | 0.464 |
|  | Interaction HT-Women | 1.09 | 0.58 | 1.60 | <0.001 | 1.77 | 1.03 | 2.51 | <0.001 | - | - | - | - |
|  |  |  |  |  |  |  |  |  |  |  |  |  |  |
|  |  | **Complete** | | | | **Younger (<50)** | | | | **Older (≥ 50)** | | | |
| BPW | **Term** | **Est.** | **95%CI** | | **p** | **Est.** | **95%CI** | | **p** | **Est.** | **95%CI** | | **p** |
|  | NT | 0.00 | Ref |  |  | 0.00 | Ref |  |  | 0.00 | Ref |  |  |
| Age, ethnicity | HT | 2.32 | 2.04 | 2.60 | <0.001 | 1.82 | 1.48 | 2.17 | <0.001 | 2.87 | 2.53 | 3.22 | <0.001 |
|  | Women | -0.36 | -0.58 | -0.14 | 0.001 | -0.72 | -0.93 | -0.50 | <0.001 | 0.49 | 0.16 | 0.82 | 0.004 |
|  | Interaction HT-Women | 0.91 | 0.54 | 1.28 | <0.001 | 1.58 | 1.09 | 2.07 | <0.001 | - | - | - | - |
| Age, ethnicity, height | NT | 0.00 | Ref |  |  | 0.00 | Ref |  |  | 0.00 | Ref |  |  |
|  | HT | 2.32 | 2.04 | 2.60 | <0.001 | 1.82 | 1.48 | 2.17 | <0.001 | 2.87 | 2.53 | 3.22 | <0.001 |
|  | Women | -0.48 | -0.76 | -0.19 | <0.001 | -0.69 | -0.98 | -0.39 | <0.001 | 0.25 | -0.23 | 0.73 | 0.304 |
|  | Interaction HT-Women | 0.91 | 0.54 | 1.28 | <0.001 | 1.58 | 1.09 | 2.08 | <0.001 | - | - | - | - |

**Supplementary table 3:**

Regression analysis showing the relationship between HT and the FPW amplitude and BPW amplitude with correction for age, sex and ethnicity in the crude model, and additional correction for height in the second model. Estimates depict association with FPW and BPW in mmHg. Interaction term depicts interaction between sex and hypertension; estimates are depicted with men as reference. (FPW = forward pressure wave, Est = estimation, CI = confidence interval, NT = normotensives, ref = reference, HT = hypertensives, BPW = backward pressure wave).

**Supplementary figure 1:** Data selection for final analysis based on quality criteria defined in.^30^

**
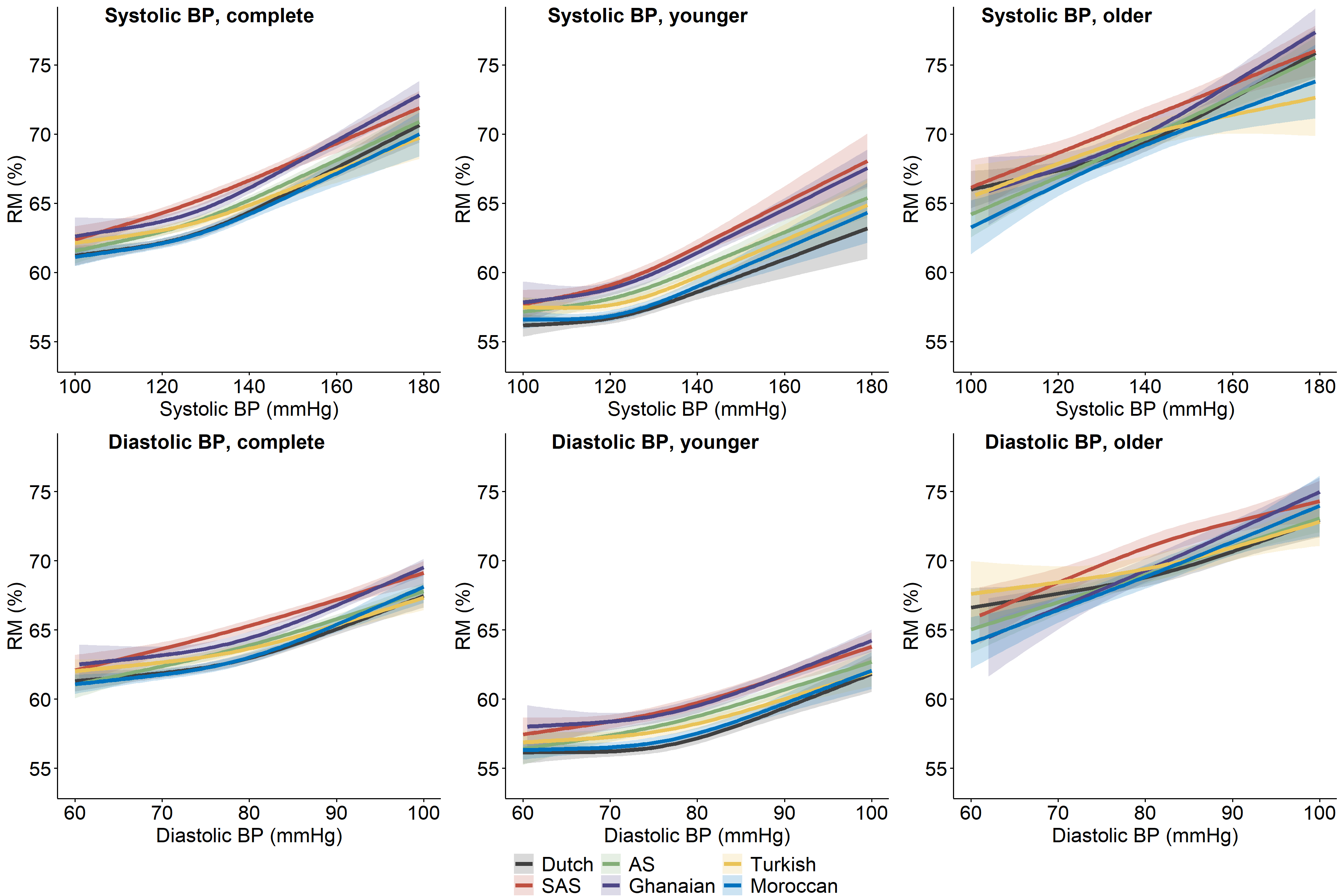
**

**Supplementary figure 2:** Relationship between systolic and diastolic blood pressure and reflection magnitude in the complete cohort, in younger (<50 years) and older (≥50 years), stratified by ethnicity. Lines represent the results from regression model with correction for age, sex, ethnicity; depicted with respect to age of 35 years in younger cohort; 65 in older cohort and 50 in the complete cohort; for men. RM denotes reflection magnitude, BP blood pressure. There was only a significant interaction with ethnicity for DBP in the older subgroup (p=0.031). (RM = reflection magnitude, BP = blood pressure, AS = African Surinamese, SAS = South-Asian Surinamese)
